# Regenerative Medicine in Organ and Tissue Transplantation: Shortly and Practically Achievable?

**Published:** 2015-08-01

**Authors:** A. Heidary Rouchi, M. Mahdavi-Mazdeh

**Affiliations:** Iranian Tissue Bank and Research Center, Tehran University of Medical Sciences, Tehran, Iran

**Keywords:** Regenerative medicine, Organ transplantation, Tissue transplantation, Multiple organ failure, Hematopoietic stem cell transplantation, Tissue engineering

## Abstract

Since the beginning of organ/tissue transplantation, the therapeutic modality of choice in end-stage organ failure, organ shortage has been the main problem in transplantation medicine. Given the so far unsolved obstacle, all hope-raising procedures to possibly tackle this long-lasting problem can draw attentions. In this context, “regenerative medicine” sounds to be more promising compared to other approaches. To consider the huge impact of hematopoietic stem cell transplantation on the treatment of some congenital or acquired hematological or metabolic disorders and some advances to produce tissue engineered materials on one hand, and to take all aspects of this emerging and costly interdisciplinary field of research into consideration, on the other hand, inevitably makes this reality unchanged, in particular in countries with low or middle income, that allograft (from deceased or living donors) will remain for years as the irreplaceable source of organ transplantation.

## INTRODUCTION

Allogenic grafts or allografts are presently irreplaceable tissue grafts to replace damaged tissues or non-functioning organs to restore their normal function. For decades, organ shortage has been the main disappointing barrier in transplantation medicine. Nevertheless, we have not yet found ways to overcome the complex issue of the shortage of organs for transplantation. Our patients still die in unacceptably high numbers on waiting list. One can imagine number of approaches for the future; however, can regenerative medicine (RM) significantly contribute to addressing organ shortage in future [1]?

Recent notable achievements in RM have aroused a lot of curiosity among transplant clinicians and researchers. RM is changing the premise of solid organ transplantation, requiring transplantation investigators to become familiar with RM investigations that can be extremely relevant to their work. Similarly, RM investigators need to be aware of needs of the transplant field to bring these two fields together for greater results [2].

This article mainly reviews the RM and tries to bring this field of research into consideration, which has brought hopes to treat a number of currently just controllable diseases. However, particular emphasis is given to the probable seat of RM as a replacement therapy in patients suffering from end-stage organ failure.

## REGENERATIVE MEDICINE

Definitions

RM replaces or regenerates human cells, tissue or organs to restore or establish normal function [3]. To define in length, RM is an emerging interdisciplinary field of research and clinical applications focused on the repair, replacement or regeneration of cells, tissues, or organs to restore impaired function resulting from any causes, including congenital defects, diseases, trauma, and aging. It uses a combination of several technological approaches that moves it beyond traditional transplantation and replacement therapies. These approaches may include, but not limited to, the use of soluble molecules, gene therapy, stem cell transplantation, tissue engineering, and the reprogramming of cell and tissue types [1, 4]. 

Over the past decade, significant advances in the fields of stem cell biology, bioengineering, and animal models have converged on the discipline of RM. In this field, the scientists use the capacity of stem cells for restoring damaged tissues by transplantable tissue patches that preserve three-dimensional structure [5]. Indeed, RM use natural human substances, such as genes, proteins, cells and biomaterials to regenerate diseased or damaged human tissue in order to restore normal function. It is believed that RM has the potential to dramatically improve our ability to fight disease and to repair the human body [6]. It combines the knowledge and skills of several disciplines towards the aim of addressing impaired function in the body. Its goal is not just to replace what is malfunctioning, but to provide the elements required for *in vivo *repair, to devise replacements that inconspicuously interact with living body and stimulate the body’s intrinsic capacities to regenerate [7].

Principles

The central focus of RM is human stem cells. A stem cell can become any cells and therefore it can potentially (and theoretically) create any tissues or organs. Under specific conditions, stem cells can differentiate into a diverse population of mature and functionally specialized cell types. To date, different pluripotent stem cell types have been described; these include embryonic stem cells, cord blood stem cells, and adult bone marrow stem cells [8]. Though inevitably the pioneering phase leading towards RM has been marked by some failures, there are now some commercial products for skin ulcer, bladder dysfunction, and sports injury damage to the cartilage of the knee. The pluripotent cells can be autologous or allogenic somatic cells and in the case of skin and bladder, the products have a biomaterial component [9].

Human Embryonic Stem Cells

Human embryonic stem cells (hESCs) have the capacity for self-renewal, long-term proliferation and pluripotency, making them a primary candidate for tissue engineering and regenerative therapies. So far, numerous hESC lines have been developed and characterized. Deriving specific cell populations from hSECs, which could either replace damaged cells or coax neighboring cells to function normally, provides a promising strategy for cell-based therapy. With hESCs it is possible to generate lineage-restricted progenitors that are capable of differentiating into post-mitotic cell types such as cardiomyocytes, pancreatic islet cells, chondrocytes, hematopoietic cells, endothelial cells, or neurons [10].

To compare, hESCs are with very high replicative potential. They also have malignant potential, and also have issues with rejection and ethical concerns. A new cell class is also described and derived from amniotic fluid and placental tissue obtained during pregnancy or at the time of birth. This system avoids the malignant potential and ethical concerns surrounding the use of hESCs. The stem cells then can be rapidly expanded to large quantities sufficient for clinical translation, thus avoiding the limitations of adult bone marrow stem cells.

Cord Blood Stem Cells

Since a person's own (autologous) cord blood stem cells can be safely infused back into themselves without being immunologically rejected, and because they have unique characteristics compared to adult bone marrow stem cells, they are an increasing focus of RM. The field of RM can be expected to benefit greatly as additional cord blood stem cell applications are researched and more people have access to their own preserved cord blood using state/private banking facilities.

Current Top-ranked Clinical Applications of Regenerative Medicine

The first-ranked application was frequently supported by reference to the high prevalence of diabetes mellitus (DM) and the resultant major health, social, and economic burdens. It is emphasized that controlling DM would in turn reduce the incidence of complications such as visual disturbance, heart disease, chronic kidney disease, and diabetic neuropathy and ulcer. The panelists noted that repeated insulin treatments are costly and inaccessible to many patients. They felt that RM therapies, such as bone marrow stem cell transplantation or microencapsulated islet cells using novel biomaterials, could increase accessibility by providing a permanent solution and reduction the financial burden [11].

Autologous cells for the regeneration of heart muscle after myocardial infarction and cardiomyopathy is second-ranked clinical application of RM. As one of the most terminally differentiated organs, the endogenous regenerative potentials of the adult heart are extremely limited and insufficient to compensate for cardiac tissue loss. Consequentially, exogenous regenerative strategies, especially cell replacement therapy, have emerged and attracted increasingly more attention in the field of cardiac tissue regeneration. Induced pluripotent stem cells, and embryonic stem cells, like cells that are derived from somatic cells by reprogramming, represent a promising candidate due to their high potentials for self-renewal, proliferation and differentiation. More importantly, they provide an invaluable method of deriving patient-specific pluripotent stem cells [12]. Autologous cells, potentially injected directly into damaged regions of the heart or used in regenerative myocardial patches, have the advantage of avoiding rejection and, hence, costly immunosuppressive regimens [11].

Tissue-engineered skin substitute using autologous stem or progenitor cells are the other materials in this field. Intelligent dressings composed of a slow-releasing growth hormone polymer are being used in skin loss conditions such as burns, wounds, and diabetic ulcers [11].

## ORGAN TRANSPLANTATION VS ORGAN SHORTAGE

Organ transplantation may be referred to as one of the greatest achievements in the history of medicine. Short-, medium- and long-term results are excellent and, when compared with patients who cannot receive a new organ, the impact on patient’s mortality, morbidity and quality of life is tremendous. If we consider kidney transplantation as a paradigmatic example, it currently represents the gold-standard for renal replacement therapy in patients affected by end-stage renal disease. In fact, when compared with maintenance peritoneal or hemodialysis, renal transplantation dramatically improves patient survival, quality of life, and is cost-effective in terms of health care expenditures. According to a United States Renal Data System report as of July 27, 2012, life expectancy from the time the dialysis is initiated is approximately eight years for patients between the ages of 40 and 44, and 4.5 years for those who are between 60 and 64 years of age. These figures are far surpassed by the increased survival rates following kidney transplantation, which are 85%, 70%, and 44% after 5, 10, and 20 years, respectively [13]. Similar data are reported by the European Renal Association-European Dialysis and Transplantation Association (ERA-EDTA) Registry, based on 2012 data, for which expected remaining life-times in years of dialysis *versus* transplant patients in the age groups of 40–44 and 60–64 years are 11.5 *vs* 25.5 years, and 5.8 *vs* 12.3, respectively [14]. The main restriction of this modality is, however, organ shortage. Based on “Organ Donation and Transplantation Activities Report in 2012,” released by Global Observatory on Donation and Transplantation (GODT) affiliated to World Health Organization (WHO), the total number of 114,690 solid organs reported to be transplanted in 2012 with just 1.8% of increase over 2011 and surprisingly less than 10% of global needs [15]. Similarly, at the end of 2011, 96,574 patients were waiting for a kidney in the USA, whereas only 16,813 kidney transplants were performed during the calendar year. This corresponds to a cumulative probability to receive a renal graft at the first year (from the time of registration on the waiting list) of only 9.65%. This figure increases at 3^rd^ and 5^th^ years to 21.65% and 36%, respectively—a rate that is not sufficient to satisfy the demand for transplantable kidneys [13].

As these figures illustrate, the greatest challenge facing the field of organ transplantation is a dramatic increase in the number of patients waiting for allografts in one hand, and the limitation of available organs for transplantation, on the other hand. Accordingly, a variety of approaches have been implemented to expand the organ donor pool including live donation [16, 17], international efforts to expand deceased donor donation (donors after brain death and donors after circulatory death), split organ donation, paired donor exchange, national sharing models, and greater utilization of expanded criteria donors [18]. Nonetheless, all of these approaches have not yet been completely successful to overcome the organ shortage and the gap between increasing demand and supply of organs is widening, the cumulative probability to receive an organ in the critical time frame is dropping drastically and the mortality on the waiting list is fearfully increasing [13].

## REGENERATIVE MEDICINE AND ORGAN TRANSPLANTATION

Regenerative Medicine as Applied to Solid Organ Transplantation

As we continue to have severe shortages of organs for transplantation, we need to consider alternatives for the future. The most likely to make a real difference in the long term is RM, a field that has emerged from the conjunction of stem cell biology and cell therapies, gene therapy, biomaterials and tissue engineering, and organ transplantation. Transplantation and RM share the same essential goal—to replace or restore organ function [19]. Recent groundbreaking advances in organ bioengineering and regeneration have provided evidence that RM holds the promise of engineering damaged tissues and organs via stimulating the body’s own repair mechanisms to functionally heal previously irreparable tissues or organs. RM also includes the possibility of growing tissues and organs in the laboratory and safely implants them when the body cannot heal itself. If a regenerated organ’s cells would be derived from the patient’s own tissue or cells, this would potentially solve the problem of the shortage of organs available for donation, and the problem of organ transplant rejection.

“Organ transplantation and RM; however, share a common heritage. Alexis Carrel can be considered the father of both RM and organ transplantation” [20, 21], and it is now clear that his legacy is equally applicable for the present and future generations of transplant and RM investigators [13]. Progress made in cell and stem cell biology, material sciences and tissue engineering enables researchers to develop cutting-edge technology, which has led to the creation of non-modular tissue constructs such as skin, bladders, vessels and upper airways. In these cases, supporting scaffolds are required to autologous cells to be seeded. Nevertheless, such constructs receive nutrients and oxygen by diffusion from adjacent tissues, because they lack vascular supply. Engineering of organs with functioning units and vascular supply is a complicated issue. These efforts using natural scaffold have been made to engineer functioning hearts, livers, kidneys, pancreata, and small intestine. However, it seems that there is a long way to go to achieve this goal in near future and in a number fulfilling the increasing needs of patients and with reasonable and affordable costs. The idea of creation of bioengineered solid organs with autologous cells and internal vasculature will optimistically relieve the main and grave concerns of organ transplantation, organ shortage and complications of life-time immunosuppressive therapy [22]. 

Transplantation with No Immunosuppressive Medications

Organ transplantation without need to immunosuppression will be an outstanding achievement in transplantation medicine. In this case, recipients are free from all complications caused by immunosuppression including, but not restricted to, infections, metabolic disturbances, malignancies, cardiovascular events, and medications’ side effects. This condition would definitely reduce the post-transplantation treatment costs and improve patients’ quality of life significantly [13]. 

About half of the renal allografts are lost within 10 years following transplantation mainly due to allogenicity and nephrotoxicity of immunosuppressive therapy. Presently, an immunosuppressive-free status is attainable once organs from genetically identical donors are used or by rarely establishment a state of immunological tolerance. Both of these situations are limited to a really few number of recipients, while the majority of them have to face with inadvertent complications of immunosuppression after transplantation. In case of liver transplantation, an immunosuppression-free state is practically more probable, compared to other organs, at least one year following the transplantation. However, this status is neither immediate, nor stable. This desirable condition is transiently effective and attainable in just 25% of recipients with planned weaning of immunosuppression. In spite of substantial advances in the context of transplantation immunology, there is not yet adequate understanding of tolerance mechanisms [13]. 

Hollow *vs* Solid Organs

So far, RM has been successful to produce hollow organs such as vessels, upper airways, urethras, and bladder which they function independently on a direct connection to the recipients’ blood supply. These tissues/organs are less complex than solid organs, which consisted of functioning units and internal vasculature and accordingly require reconnection to the recipients’ vascular system at the time of implantation. Still, bioengineering of cardiac, hepatic, renal, pancreatic, and intestinal organ-like constructs is not practically possible. Currently, the technology, namely cell-scaffold technology, being used for organ bioengineering is the seeding of pluripotent stem cells on supporting structures. In human body, extracellular matrix plays an important role as this supporting frame. Growth factors are other essential elements of this process [13]. 

Vascularization

Notably, all bioengineered organs that to date have been implanted successfully have been hollow organs in which reconnection to the systemic vascular system is not necessary. In case of solid organs, the scenario differs absolutely, where connection to the vascular system is a must. Cells can survive merely within 1–3 mm away from a source of nutrients and oxygen. Aiming to connect the solid organs to systemic circulation, natural extracellular matrix scaffolds obtained from the decellularization of organs are the perfect platforms owning to their ability to maintain a properly preserved framework of their intrinsic vasculature [13].

## CONCLUSION

Despite the advances of RM in providing a number of tissues for transplantation, in particular the outstanding success in use of hematopoietic stem cells in treatment of some blood disorders, this promising field has not yet been fully successful to provide organ for transplantation mainly due to natural complexity of solid organs. Still, and maybe in near future, human organs will be the only source for transplantation. More prominently in developing countries and all other countries with low or middle income, considering the costly procedures of these kinds of research, it makes sense to think firstly to establish a new system or expand the existing practice on organ procurement from deceased donors to supply sufficient, safe and optimal grafts in response to an ever-increasing demand for transplantable organs [23].

## Notes

**FINANCIAL SUPPORT:** None declared.
